# Supplementary material for: Physicochemical Characterization of Kynurenine Pathway Metabolites
Source: Antioxidants (Basel). 2025 May 14;14(5):589. doi: 10.3390/antiox14050589 (PMC12108296; doi:10.3390/antiox14050589)
Supplement: Supplementary file 1 [file antioxidants-14-00589-s001.zip › chemical calculations/Kynurenic acid calculations.docx]

L-Kynurenine conformers MacroModel conf. search

| **Conformer #** | **Potential Energy**  **(kJ/mol)** | **Rel. Pot. Energy**  **(kJ/mol)** |
| --- | --- | --- |
| 1 * | -504.494 | 0.000 |
| 2 | -502.962 | 1.532 |
| 3 | -498.602 | 5.892 |
| 4 | -498.332 | 6.162 |
| 5 | -497.582 | 6.913 |
| 6 | -496.727 | 7.768 |
| 7 * | -495.713 | 8.781 |
| 8 | -495.325 | 9.169 |
| 9 | -492.445 | 12.049 |
| 10 | -491.036 | 13.458 |
| 11 | -490.694 | 13.801 |
| 12 | -490.241 | 14.253 |
| 13 | -490.067 | 14.428 |
| 14 | -489.575 | 14.920 |
| 15 | -489.411 | 15.084 |
| 16 | -488.224 | 16.271 |
| 17 | -487.919 | 16.575 |
| 18 | -487.522 | 16.972 |
| 19 | -487.239 | 17.256 |
| 20 | -485.873 | 18.622 |
| 21 | -484.326 | 20.168 |
| 22 | -483.960 | 20.534 |
| 23 | -483.544 | 20.951 |

3-Hydroxykynurenine conformers MacroModel conf. search

| **Conformer #** | **Potential Energy**  **(kJ/mol)** | **Rel. Pot. Energy**  **(kJ/mol)** |
| --- | --- | --- |
| 1 * | -382.503 | 0.000 |
| 2 | -382.078 | 0.425 |
| 3 | -380.856 | 1.647 |
| 4 | -380.283 | 2.220 |
| 5 | -376.357 | 6.146 |
| 6 | -375.675 | 6.829 |
| 7 | -375.626 | 6.877 |
| 8 | -375.123 | 7.380 |
| 9 | -374.657 | 7.846 |
| 10 | -374.603 | 7.900 |
| 11 | -374.042 | 8.461 |
| 12* | -373.612 | 8.891 |
| 13 | -372.923 | 9.581 |
| 14 | -372.598 | 9.905 |
| 15 | -371.673 | 10.830 |
| 16 | -370.919 | 11.584 |
| 17 | -370.655 | 11.848 |
| 18 | -368.860 | 13.644 |
| 19 | -368.721 | 13.782 |
| 20 | -368.231 | 14.272 |
| 21 | -368.128 | 14.375 |
| 22 | -367.839 | 14.664 |
| 23 | -367.546 | 14.957 |
| 24 | -367.380 | 15.123 |
| 25 | -367.075 | 15.429 |
| 26 | -367.034 | 15.469 |
| 27 | -366.471 | 16.032 |
| 28 | -366.287 | 16.216 |
| 29 | -366.280 | 16.223 |
| 30 | -365.894 | 16.609 |
| 31 | -365.699 | 16.804 |
| 32 | -365.644 | 16.859 |
| 33 | -365.546 | 16.957 |
| 34 | -365.310 | 17.193 |
| 35 | -365.126 | 17.378 |
| 36 | -364.714 | 17.789 |
| 37 | -364.196 | 18.308 |
| 38 | -363.292 | 19.212 |
| 39 | -362.732 | 19.771 |
| 40 | -362.392 | 20.111 |
| 41 | -362.131 | 20.372 |
| 42 | -361.798 | 20.705 |

L-Kynurenine conformers (MacroModel conf. search

|  | **Gas Phase Energy**  **(Hatree)** | **Rel. GPE**  **(Hatree)** | **Solution Phase Energy**  **(Hatree)** | **Rel. SPE**  **(Hatree)** |
| --- | --- | --- | --- | --- |
| conf 1* | -723.473103 | 0 | -723.556838 | 0 |
| conf 7* | -723.486035 | 0.012932 | -723.558207 | 0.001369 |

3-Hydroxykynurenine conformers MacroModel conf. search

|  | **Gas Phase Energy**  **(Hatree)** | **Rel. GPE**  **(Hatree)** | **Solution Phase Energy**  **(Hatree)** | **Rel. SPE**  **(Hatree)** |
| --- | --- | --- | --- | --- |
| conf 1* | -798.690155 | 0 | -798.781032 | 0 |
| conf 12* | -798.703440 | 0.013285 | -798.782693 | 0.001661 |
